# Lying-In

**Published:** 1894-12-22

**Authors:** 


### LYING-IN.

**The City of London Lying-in Hospital**, City Road, E.C.—The number of patients at this hospital again shows an increase, over 2,200 poor women having been delivered either in the hospital or at their own homes, and that with the most satisfactory results, only one maternal death in every 1,200 deliveries having taken place in the hospital since May, 1891. This fact is a sufficient proof of the care and attention bestowed upon the patients, and of the antiseptic treatment, which is fully carried out. The committee have enlarged the hospital during the past year by building additional dormitories, and a dining-hall for the pupils and nurses, at a cost of over £2,000. The committee earnestly appeal to the public for £1,500 to repay loans to bankers and to enable them to carry on the general work of the hospital. Secretary, Mr. R. A. Owthwaite.

